# Peripheral Visual Cues: Their Fate in Processing and Effects on Attention and Temporal-Order Perception

**DOI:** 10.3389/fpsyg.2016.01442

**Published:** 2016-10-06

**Authors:** Jan Tünnermann, Ingrid Scharlau

**Affiliations:** Faculty of Arts and Humanities, Psychology, Paderborn UniversityPaderborn, Germany

**Keywords:** cueing, temporal-order judgements, theory of visual attention (TVA), peripheral cue, processing speed, stimulus encoding, prior entry

## Abstract

Peripheral visual cues lead to large shifts in psychometric distributions of temporal-order judgments. In one view, such shifts are attributed to attention speeding up processing of the cued stimulus, so-called prior entry. However, sometimes these shifts are so large that it is unlikely that they are caused by attention alone. Here we tested the prevalent alternative explanation that the cue is sometimes confused with the target on a perceptual level, bolstering the shift of the psychometric function. We applied a novel model of cued temporal-order judgments, derived from Bundesen's Theory of Visual Attention. We found that cue–target confusions indeed contribute to shifting psychometric functions. However, cue-induced changes in the processing rates of the target stimuli play an important role, too. At smaller cueing intervals, the cue increased the processing speed of the target. At larger intervals, inhibition of return was predominant. Earlier studies of cued TOJs were insensitive to these effects because in psychometric distributions they are concealed by the conjoint effects of cue–target confusions and processing rate changes.

## Introduction

Attention is believed to selectively speed up processing. The resulting effect is known as “prior entry,” the earlier perception of a stimulus to which attention is directed compared to an unattended one, all else being equal. The effect had already troubled eighteenth-century astronomers, whose judgment of visual events relative to an auditory time reference was involuntarily affected by the distribution of their attention (Hoffmann, 2007; Vatakis, 2013). Titchener (1908) included this effect in his fundamental laws of attention.

Nowadays, prior entry is frequently investigated with temporal-order judgments (TOJ; e.g., Shore et al., 2001; Scharlau et al., 2006; Weiß et al., 2013; Schettino et al., 2016). TOJs are collected in experiments in which participants report the order of two stimuli. The asynchrony in target presentation is varied. The data is typically analyzed by fitting psychometric functions and estimating parameters, such as the point of subjective simultaneity (PSS) and the difference limen (DL; Woodworth and Schlosberg, 1954; Wichmann and Hill, 2001). Attending to one of the stimuli changes the PSS in favor of this stimulus. The DL parameter is an estimate of the discrimination performance. Importantly, these parameters describe the observed judgments but not the processes that lead to them. Consequently, this method cannot describe the mechanisms that cause prior entry.

Peripheral cues are common to direct attention in TOJs, and they elicit substantial shifts of the PSS (Shore et al., 2001). However, up to now, when modeling TOJs, cues have only been considered as an influence on attention, not different from an instruction despite that they cause much larger effects. Is a cue not a stimulus of its own, whose fate in processing and potential encoding has to be considered? We argue that this is the case, and show that including concrete stimulus processing models for the two targets and the cue changes the picture of cued TOJs entirely. This approach can explain why the effect of peripheral cues on the PSS is so suspiciously strong. Furthermore, the results explain why the expected inhibition of the target location after a long cueing interval, known as inhibition of return (Klein, 2000), has not been observed in cued TOJs until now (Scharlau et al., 2006).

The model of stimulus encoding we employ is based on Bundesen's Theory of Visual Attention (TVA; Bundesen, 1990; Kyllingsbæk et al., 2015). In TVA, stimuli race for a slot in visual short-term memory (VSTM). Processing of a stimulus starts as soon as it is presented for longer than a threshold, *t*_0_, and encodings occur according to a rate parameter *v*. A model that takes theses races into account for all stimuli, including the cue, is the basis for this article. After this anticipation of the agenda of this study, we return now to the fundamental limitations of traditional TOJ analysis.

Because of the limited meaning of the summary parameters, PSS and DL, which are usually estimated, it was unclear until recently whether prior entry in TOJs arises from speeding up processing of the attended stimulus or slowing down processing of the unattended one. TOJs are fundamentally relative. That is, without modeling the encoding processes that cause them, acceleration of the attended and deceleration of the unattended stimulus cannot be distinguished from TOJ data alone. Weiß et al. (2013) and Tünnermann et al. (2015) showed that a decrease of the processing speed of the unattended stimulus indeed contributes substantially to prior entry. These experiments, however, included a difficult dual task, in which the observers provided TOJs and a secondary response that allowed measuring the latencies of both targets individually. In Tünnermann et al. (2015), with target, cue and mask onsets, the latter required by the secondary letter-report task, the presentation was quite complex. The results showed that prior entry was more than twice as large when measured with TOJs compared to the secondary task. Furthermore, the TOJ-based prior entry increased with the cueing interval, whereas it remained the same at different intervals when measured via the secondary task. Most likely, differences in the tasks, for example that the temporal onset signals of the cue and the masks may interfere with the TOJ but not the secondary task, could have driven this discrepancy. Therefore, it is necessary to study the speedup vs. slowdown question within the TOJ task, taking the cue into account as a proper stimulus.

Peripheral cues are known to effectively direct attention in TOJs (Shore et al., 2001). Their ability to capture attention and facilitate processing is also well known outside the TOJ domain. Carrasco and McElree (2001), for example, showed that covert attention directed by peripheral cues not only enhances the discriminability of targets but also increases their processing rate. The similarity between the cue and the targets plays an important role in research on contingent capture. Contingent-capture studies investigate how the ability of task-irrelevant stimuli to capture attention depends on the relationship between the stimulus and the properties required for the task (see Folk et al., 1992). It has been demonstrated that peripheral onset signals capture attention automatically even if they are irrelevant for the task (Remington et al., 1992). The exact boundary conditions under which attentional capture is chiefly automatic or when it is contingent on the observer's goals are subject of ongoing discussions (e.g., see Ansorge et al., 2010; Theeuwes et al., 2010). It is probably safe to say that similarity of a visual cue with targets, or relevancy for the task due to other factors, increases its effectiveness in directing attention. In TOJs, cues have onsets, just like the targets whose order is to be judged. Onset time is a particularly important feature for order discrimination. Often further attributes such as color or shapes are shared between cue and target. Therefore, it is not surprising that peripheral cues guide attention in TOJs. It is, however, very curious how strong the effects of peripheral cues are in TOJs.

Typically, large shifts in the PSS are found (Shore et al., 2001; Scharlau and Neumann, 2003). One could even say their effects are conspicuously large. They are larger than those of other attention manipulations (Shore et al., 2001; Schneider and Bavelier, 2003; Tünnermann et al., in press), and it is difficult to explain such large shifts by influences on the processing speed theoretically. TVA (Bundesen, 1990), for instance, needs to be complemented with parameters for additional delays which have no theoretical justification to fit TOJ data (Tünnermann et al., in press). Furthermore, the time course of prior entry in cued TOJs is rather untypical. The effect remains positive even at large cueing intervals where inhibition of return (IOR; Klein, 2000) would be expected (cf. Scharlau et al., 2006).

The conspicuities of peripheral cueing in TOJs require explanation. Schneider and Bavelier (2003) suggested that the cue may induce additional non-attentional effects or that it sometimes is confused with the target stimulus. Pashler (1998) mentioned similar suspicions that cue and target may become an amalgamated complex or are confused at a perceptual level (p. 260). The tentative confusion explanation fits well in the theoretical framework of TVA. According to TVA, every stimulus races for every possible categorization. Normally, sensory evidence and category biases are practically zero for non-targets. Therefore, it can be safely ignored that a particular shape that is shown in a TOJ races with some close-to-zero rate for being categorized, for example, as a pianoforte. However, the peripheral cue in a TOJ has certain target attributes. Most importantly, it has an onset signal, an attribute important in TOJs. Hence, Schneider and Bavelier's as well as Pashler's tentative explanation agree quite well with TVA. Similar to the relation between cue and target attributes, which influences the ability of the cue to direct attention (as in the contingent-capture research mentioned above), according to TVA, it is relevant that the cue can be encoded as a target. Whether this really explains the effectiveness of peripheral cues for shifting the PSS is tested in the present study.

To put it precisely, the present study tests the hypothesis that prior entry in cued TOJs arises mainly from confusing the cue with the cued target. We apply an advanced TVA-based TOJ approach which models confusions as “cue is cued target” categorizations. The model includes further parameters for the processing speeds of both targets. It can therefore reveal whether processing rate changes are present, as suggested by the prior-entry research that used secondary tasks. Importantly, the model can be applied to data from simple TOJs without the requirement of additional tasks or visual events such as masking stimuli.

We conducted two experiments to test this hypothesis. In Experiment 1, the interval between the cue and the target is varied to test the prediction that broad shifts of psychometric functions result from cue–probe categorizations with no or only moderate changes in the processing rates of the targets. With increasing time available for the cue to be encoded as probe, this event becomes more likely to happen for a given trial, explaining the increase of the PSS shift which depends on the cueing interval. Experiment 2 tests a further prediction: When the cue is gradually moved away from the target position, it loses the co-locality attribute, which should decrease its rate as being encoded as the cued target and possibly affect the target processing rates.

Before describing the experiments and their results, the following sections explain the model that was used to analyze the data. In describing the model and in the remainder of this article, we use some terminology. We will refer to the cued target as “probe” and to the uncued target as “reference.” The reference is always shown at a virtual time zero, with the probe being shown relative to it according to the SOA (stimulus onset asynchrony) ranging from negative (probe before reference) to positive (reference before probe) values. At SOA zero, both targets are shown simultaneously. The cueing interval will be called “cue onset asynchrony” (COA) in the following.

**Figure 1:**
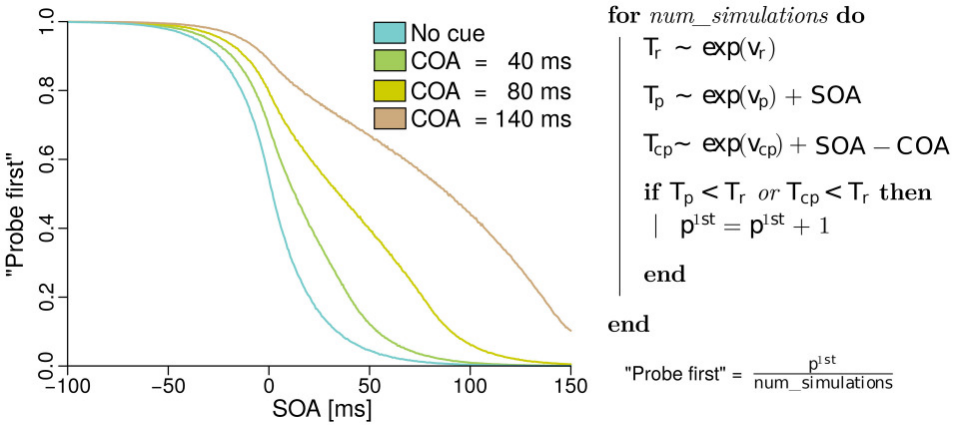
**Simulated psychometric distributions of “probe first” temporal-order judgments over a range of SOAs (stimulus onset asynchronies) with rates ***v***_***p***_ = ***v***_***r***_ = 50 Hz and ***v***_***cp***_ = 10 Hz**. In this hypothetical version, the rightwards shift with increasing COA (cue onset asynchrony) is generated entirely by sometimes encoding the cue as the probe target. All processing rates, including the one associated with cue–probe confusion, stay constant in this example (best viewed in color).

In the next section, the model of the probabilities for judging “probe first” depending on the SOA, COA, and the processing rates of probe, reference, and cue, derived from basic TVA equations is discussed. The reader may skip the formal derivation of the analytical model, which can be found in the Supplementary Material “Deriving probe first probabilities for cued TOJs from TVA,” without loss of continuity. However, we encourage a brief review of the simulation of the model shown in Figure 1. The simulation is described on the right of Figure 1. For every simulated trial, the probabilistic encoding times are drawn from an exponential distribution with the stimulus processing rate as the rate parameter. Hence *T*_*r*_, for example, is the time at which the reference stimulus is encoded when it races with rate *v*_*r*_. Similarly, probabilistic encoding times *T*_*p*_ are drawn for the probe and *T*_*cp*_ for the cue. For the probe, the SOA is added to the encoding time, because this is the delay that separates the starting times of probe and reference encodings. For the cue, the SOA is added and the COA subtracted, because these delays separate the cue and reference presentation times. A “probe first” percept *p*^1*st*^ is counted when cue or probe (or both) have been encoded before the reference stimulus (*T*_*p*_ < *T*_*r*_ or *T*_*c*_ < *T*_*r*_). After simulating all trials (100000 at each of 500 SOAs in the example) the *p*^1*st*^ count is divided by the number of simulations performed. This results in the relative frequencies which constitute the psychometric functions in the figure. These functions were generated with equal rates *v*_*p*_ = *v*_*r*_ = 50 Hz, and with *v*_*cp*_ = 10 Hz. Hence, the observed shift of the psychometric functions is caused solely by the mechanism of sometimes encoding the cue as the probe. When we fit this model to data, however, the rates are not restricted to equal values, to capture potential changes in the processing rates.

The model just presented in the form of a simulation captures the core idea while making two minor simplifications. The version which we fit to the experimental data differs in the following aspects: First, it accounts for the fact that the probe target masks the cue on its appearance. That is, no cue–probe confusions can appear after the probe is shown. Accounting for this leads to minute changes in the curves but complicates the simulation algorithm. Second, when the reference stimulus is shown before the cue (at large positive SOAs), it cannot be influenced by the cue and therefore receives half of all available resources, the same resources as in the neutral “no cue” condition.

The first aspect follows directly from TVA, which assumes that processing terminates when the stimulus is masked. The second one is a logical consideration. The only plausible alternative to providing the reference stimulus with half of the available resources (that is, anticipating the second target) would be that the reference obtains all available processing resources. This, however, does not fit the data well.

## A TVA-based model of cued TOJs

Traditional psychometric functions for TOJs are fitted to one type of judgment, for example, the “probe first” judgments. The same goes for the outlined model-based approach. A TVA-based simulation of our hypothesis that the cue is sometimes encoded as the probe was presented above. To test the hypothesis, an analytical form is advantageous. It can be fitted to “probe first” judgments summarized over trials like ordinary psychometric functions.

We derived such a function in Supplementary Material “Deriving probe first probabilities for cued TOJs from TVA.” The result is a function of the following form

(1) $$\begin{array}{l} P\left(p^{1st}|v_{p},v_{r},v_{cp},SOA,COA\right), \end{array}$$

where *v*_*p*_ and *v*_*r*_, are the processing rates of probe and reference and *v*_*cp*_ is the rate of cue–probe categorizations. Further arguments are the SOA and COA. This function returns the success rate of judging “probe first.” At every SOA, the responses are binomially distributed with this success rate.

To evaluate our data, we applied a hierarchical Bayesian model (Kruschke and Vanpaemel, 2015) which employs the described function. It is used to estimate the parameters for different conditions on the individual subject level and on the group level. The hierarchical model is described in Supplementary Material “A hierarchical Bayesian model of cued TOJs.” The model used in the evaluations allows for both attention-modulated target rate effects and cue–probe categorization rate effects. Hence, it can be used to measure the roles of both potential mechanisms in prior entry. A model comparison with simpler versions of the model, which only allow for either target rate effects or cue–probe categorization effects, is reported in Supplementary Material “Model comparison.”

## Experiment 1

The first experiment tests the hypothesis that the broad shifts of PSSs in TOJs at different COAs result purely from cue–probe categorizations. For this purpose, a neutral condition without cue and three experimental conditions with different COAs (40, 80, and 140 ms) are included. The different COAs are expected to cause nonzero rates *v*_*cp*_ for cue–probe categorizations, probably with magnitudes similar to the simulation (Figure 1). The probe and reference processing rate, *v*_*p*_ and *v*_*r*_ are not expected to vary substantially according to the hypothesis that prior entry in TOJs is chiefly driven by cue–probe confusion.

### Methods

#### Participants

Thirty participants (17 females, average age *M* = 22.59, *SD* = 2.41) took part in the experiment. A brief vision test was conducted to ensure that all participants had normal or corrected-to-normal vision.

#### Stimuli

**Figure 2:**
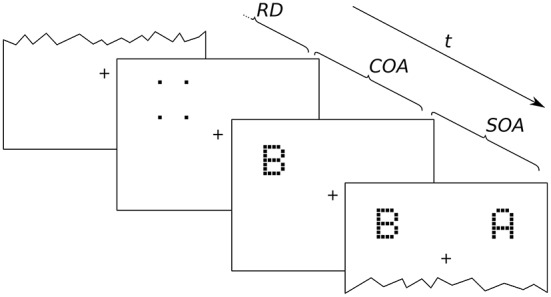
**Exemplary presentation sequence**. After a random delay (RD; 300–500 ms) cue and targets follow in the order prescribed by the SOA (stimulus onset asynchrony; here negative) and COA (cue onset asynchrony). The alternative orders are discussed in the main text.

Twenty-one letters (A, B, C, D, E, F, G, H, J, K, L, M, N, O, P, R, S, T, U, W, X), excluding easily confusable ones, were used for the probe and reference stimuli. The letters were displayed in a font originally employed by Lunau and Olivers (2010). Examples are shown in Figure 2. The letters are composed of black squares on a 5 × 7 grid and have an approximate extension of 0.8° × 1.3° of visual angle. The stimuli were reused from earlier experiments because they were known to work well with the cue. In general, almost arbitrary stimuli can be used for TOJs (Krüger et al., 2016; Tünnermann et al., in press), but using letters facilitates entering the response to proceed quickly through many trials. The cue consists of four black squares adjacent to (but not touching) the four corner cells of the target stimulus grid. The stimuli were presented on a bright gray background.

#### Presentation and response

Supported by a chin rest, the participants observed a 21″ CRT monitor from 59 cm distance. The trials were presented time-accurately via a custom-made plugin (Tünnermann et al., in press) for the OpenSesame experiment builder (Mathôt et al., 2012) at a resolution of 1024 × 768 pixels. Participants were instructed to fixate the fixation mark during the trials.

An exemplary presentation sequence is shown in Figure 2. Each trial starts with the presentation of a central fixation mark. After a random delay (300–500 ms) the first stimulus is shown. Which stimulus is presented first depends on the SOA and COA. Figure 2 shows a negative SOA. The cue is shown first and after the COA followed by the probe. After a further delay, |SOA|, the reference target is shown. If the SOA is zero, the order is the same, except that probe and reference appear simultaneously. If the SOA is positive, two further cases have to be distinguished. At SOAs smaller than the COA, the cue leads, followed by the reference stimulus after a delay of (*COA*−*SOA*). The probe stimulus follows after a further delay, the COA. Finally, if the COA is smaller than the SOA, the reference stimulus is shown first, followed after (*SOA*−*COA*) by the cue, and after a further COA delay, the probe is shown. In short, the SOA describes the relative delay between probe and references. Negative SOAs mean that the probe comes first. The COA is the delay between cue and probe.

The SOAs are shown in the header of Table 1. In every condition, nine SOAs were realized, and the distribution of the repetitions was biased so that most of the trials were close to the PSS, which, as discussed in the Introduction, shifts with the COA. The purpose of the biased distribution was to reduce the trials at the outer negative or positive SOAs, where the counted responses are typically at the ceiling and floor, respectively. Such trials are less informative than those that produce less extreme data. The number of times each SOA was repeated is shown in the table body for the different conditions in Table 1. The COAs are stated in the first column of the table. Note that trials from the different conditions were presented in an intermixed fashion.

**Table 1:** **Distribution of trial repetitions across the SOAs in the different conditions**.

**SOAs**	**−120**	**−90**	**−60**	**−30**	**0**	**30**	**60**	**90**	**120**	**150**	**180**
No-cue Cond.	4	12	20	28	32	28	20	12	4	0	0
COA = 40	0	4	12	20	28	32	32	20	8	4	0
COA = 80	0	4	8	12	20	28	32	28	20	8	0
COA = 140	0	0	4	4	12	16	24	32	32	24	12

*Each row in the table body contains nine SOAs (stimulus onset asynchronies) with nonzero counts, which have been realized for the respective conditions. SOAs and COAs (cue onset asynchronies) are in milliseconds*.

For each trial, one out of the four possible probe positions ([4°,4°],[−4°,4°],[−4°,−4°],[4°,−4°] visual angle) was chosen at random. A position for the reference stimulus was chosen at random among the remaining three positions. The cue was shown at the same position as the probe (see **Figure 6**) and erased from the screen after the COA, when the probe stimulus was switched on.

The participants entered their order judgment by typing the letters on a standard computer keyboard. The letters were displayed below the fixation mark. Participants could enter the letters in the observed order and confirm with the “Enter” key. A second stroke of the “Enter” key started the next trial. Before the second press, the “Tabulator” key could be used to invert the order judgment. Hence, it was possible to enter the letters in any order and adjust the order judgment afterward. However, the most time-efficient way of reporting the order, which the participants used most of the time, was to simply enter the letters in the order to be reported.

### Results

The goal of the model-based approach is to estimate TVA parameters. These can then be used to test which factors lead to the broad shifts observed in psychometric distributions. The data was fitted as follows.

We estimated the parameters with a hierarchical Bayesian model which encapsulates the model function (Equation 1; Equations A1–A3) in a statistical framework. With this approach, the parameters of interest, the main processing rates of probe and reference (*v*_*p*_ and *v*_*r*_) and the rate of encoding the cue as probe (*v*_*cp*_) can be estimated on the group- and subject-level. In the following, the group level estimates are marked by a μ superscript ($v_{p}^{\mu }$, $v_{r}^{\mu }$, $v_{cp}^{\mu }$). Details of the statistical model can be found in Supplementary Material “A hierarchical Bayesian model of cued TOJs.”

A first insight into the contributions of attention-modulated rate effects and cue–probe confusions can be gained by a formal model comparison between nested versions of the model that contain either mechanism alone and one which contains both of them. The details of the comparison are provided in Supplementary Material “Model comparison.” As an important result of the comparison, our hypothesis that cue–probe confusions drive prior entry must be attenuated. The comparison reveals that the full model, which contains both attention-modulated rate effects and cue–probe confusions, best describes the data (see Figure A2A). However, the particularly weak performance of the simple model without cue–probe confusions in the *COA* = 140 ms condition, where the largest PSS shifts are observed, indicates that this effect is an important contribution to the net prior-entry effect.

**Figure 3:**
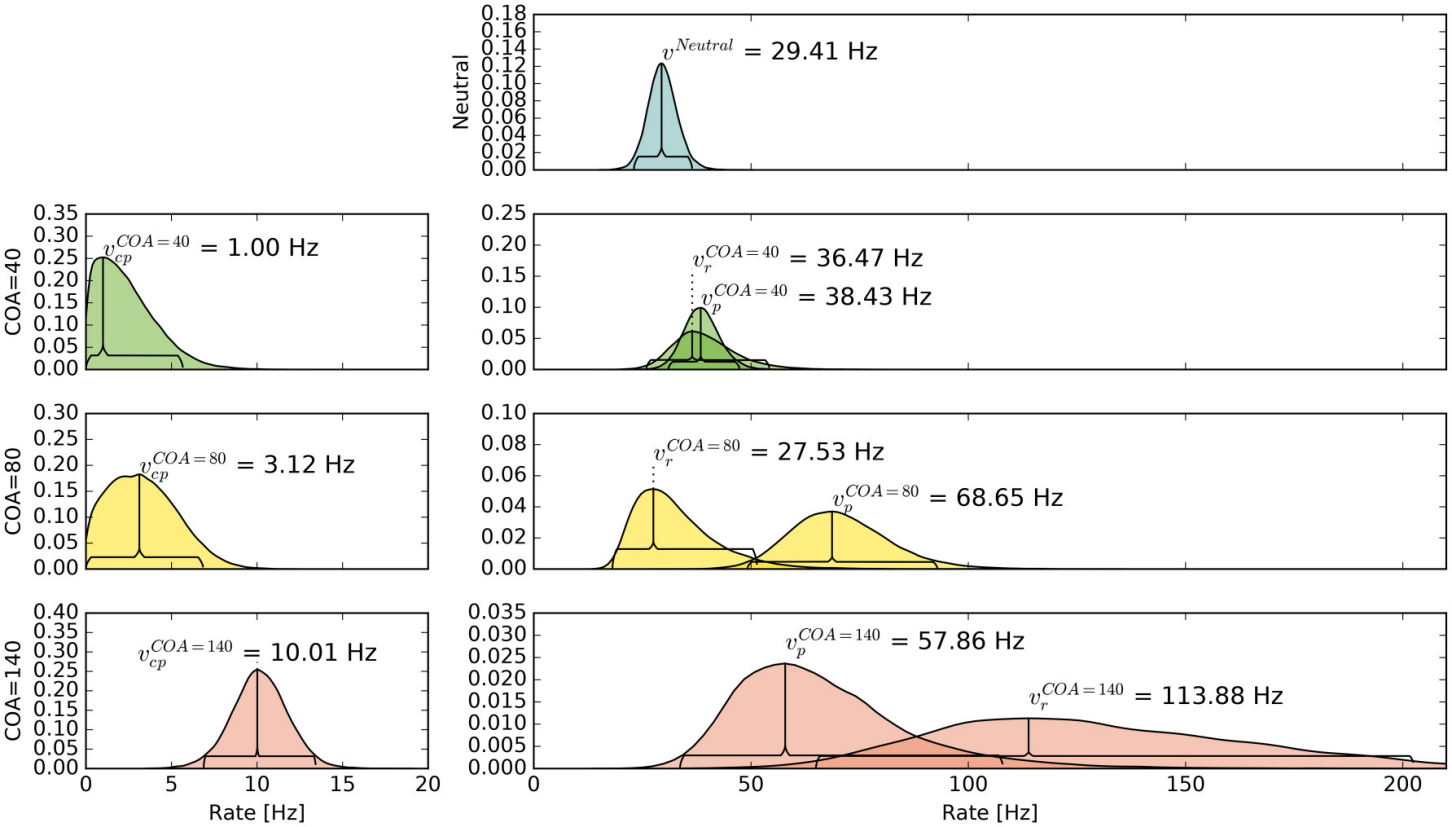
**Bayesian parameter estimates for Experiment 1**. Each row contains group-level posterior density plots of the rate parameters *v* from the different COA (cue onset asynchrony) conditions. Horizontal curly brackets mark the ranges of the 95% highest density intervals (best viewed in color).

The pattern in which attention-modulated target encoding rate changes and the cue–probe confusions contribute to prior entry at the different COAs is illustrated in the following. Posterior densities of the group-level estimates obtained with the full model are visualized in Figure 3.^1^

**Figure 4:**
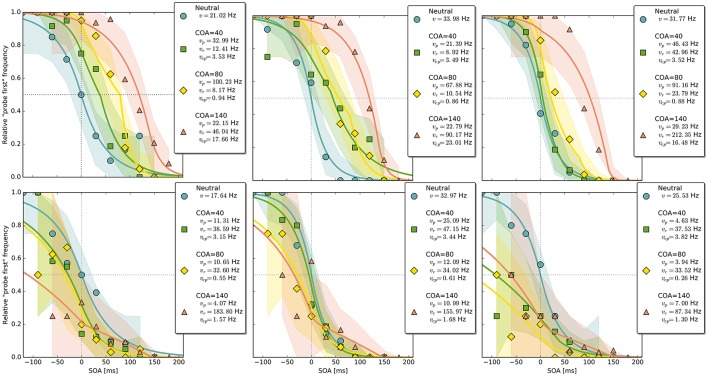
**Exemplary subject-level posterior predictive estimates and raw data points**. Solid curves are predictions obtained by using the parameter estimates. The shaded areas are based on posterior-predictive samples of the “probe first” count at each SOA (stimulus onset asynchrony). The lower row contains examples of the disadvantageous patterns which were excluded in the main group-level analysis. Data and predictions have both been normalized into the range from zero to one (best viewed in color).

In the neutral no-cue condition, the processing rate of probe and reference stimuli was estimated at 29 Hz. For *COA* = 40 ms, no reliable cueing effect was found on the group level. The *COA* = 80 ms condition shows effective cueing, which raised the rate of the probe stimulus, $v_{p}^{\mu }$, to values distributed around 69 Hz. This rate is reliably different from $v_{r}^{\mu }$. The 95% highest density interval (HDI) of the difference ($v_{p}^{\mu }-v_{r}^{\mu }$)—not depicted—does not include zero; the lower HDI boundary is at 4 Hz. At *COA* = 140 ms, the effect is reversed. The probe rate is distributed around 58 Hz, and the reference rate is increased to 113 Hz. The 95% HDI of the difference [$v_{r}^{\mu }-v_{p}^{\mu }$] does not include zero; the lower boundary is at 29 Hz. Normally, if the reference rate is higher than the probe rate, as in this case, the psychometric function would be shifted to the left. This does not happen here, it is shifted widely to the right (see upper row of Figure 4). That the reference rate can overpower the probe rate so substantially and that the model can still account for psychometric curves with a far shift to the right is due to the contribution of the cue–probe categorizations. Their rate is estimated at $v_{cp}^{\mu }=10$ Hz, zero not included in its 95% HDI, in the *COA* = 140 ms condition.

### Discussion

The group-level pattern shows that the beneficial influence of the cue on probe processing peaks somewhere around the 80 ms COA. At the 40 ms COA the effect may be small and potentially hidden on the group-level by a correlation (*r* = −0.58) between (*v*_*p*_ − *v*_*r*_) and *v*_*cp*_. Beneficial cueing effects for (*v*_*p*_ − *v*_*r*_) can be observed on the subject-level for several participants (see Figure 4 for examples). After being strong at the 80 ms COA, the positive effect on (*v*_*p*_ − *v*_*r*_) decreases and is overpowered by a strong boost of the reference rate *v*_*r*_. This reversal may by a manifestation of IOR (inhibition of return; Klein, 2000). In IOR, at long COAs attention is disengaged from the cued location before the target appears. In the present case, this leads to a probe encoding rate which is smaller than the one of the reference stimulus. A delay of 140 ms is rather short for IOR to occur. The effect is often found only after a few hundred milliseconds. However, Danziger and Kingstone (1999) report that under conditions where the cue predicts that a target can be found at a different location, IOR occurs early (even at 50 ms in their study). In our TOJs, most of the SOAs were much shorter than 140 ms and the neutral condition did not include a cue. This may have encouraged participants to disengage attention early after recognizing the first stimulus in expectation of the second stimulus, leading to relatively early IOR.

**Figure 5:**
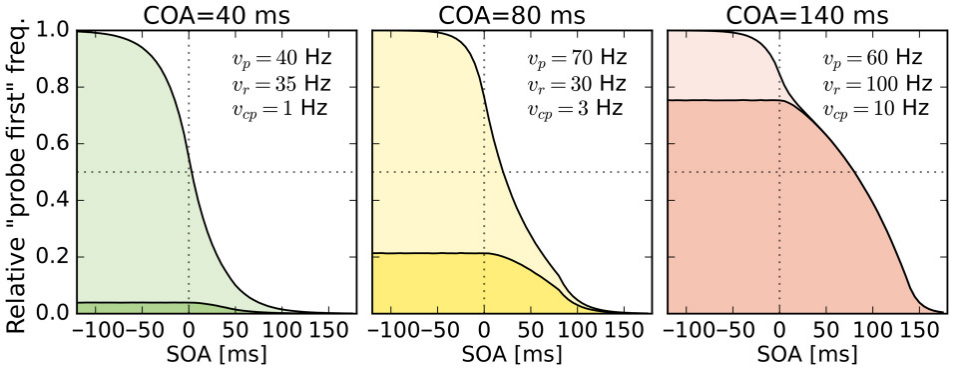
**Psychometric distributions of TOJs simulated with parameters that follow the pattern observed in Experiment 1 (cf. rates shown here and in Figure 3)**. The contributions of cue–probe categorizations to the “probe first” count are indicated by the darker shaded areas. SOA, stimulus onset asynchrony; COA, cue onset asynchrony.

Despite this disadvantageous effect on the probe at the 140 ms COA, the psychometric functions are still widely shifted in the normal direction. This is because occasionally encoding the cue as probe indeed contributes to the large shifts of the psychometric functions (see Figure 4, top row). However, at least at *COA* = 80 ms, there are genuine processing speed increases of the probe target, and at *COA* = 140 ms the reference rate is increased. That is, in contrast to our hypothesis that the whole PSS shift is produced by cue–probe confusions, processing rate changes play an important role, too. Interestingly, the advantage of the reference target at the large SOA, which normally would shift the function in the opposite direction, is concealed by the cue–probe confusions, if one merely looks at the psychometric functions and their PSS (see examples in Figure 4). Figure 5 visualizes TOJ curves of a simulated participant with values that follow the pattern revealed in Experiment 1. The contributions of probe–cue confusions are indicated in the figure.

Several important aspects, however, are not yet explained with this simple view. The overall processing rate *C* (obtained by summing up all *v* rates within a condition) is not equal across conditions. The overall rate is 58 Hz for the no-cue condition. For the other conditions it increases with COA: 74, 96, and 172 Hz. TVA's general bookkeeping of processing resources in spatially and temporally distributed paradigms has not been fully worked out yet (see Petersen et al., 2012, for how it can be modeled in a particular case). In the present experiment, the large increases in the overall rate, which depend on the COA, may be due to the non-specific activation of additional processing resources. A more detailed interpretation of how resources are distributed will be outlined in the General Discussion, taking the results of Experiment 2 into account, too.

Another interesting observation is the increase of $v_{cp}^{\mu }$ with the COA. Note that as illustrated in the simulation shown in Figure 1, PSS shifts that increase with the COA can originate from constant cue–probe confusion rates *v*_*cp*_. The increase observed here is most likely caused by varying degrees of interference from the targets. In presentation sequences in which the cue is not the first stimulus, the reference stimulus could direct attention away from it (e.g., see Weiß and Scharlau, 2011). Such presentation sequences occur more often at short than large COAs.^2^

Up to now, the results were discussed on the group level. Reviewing subject-level posterior predictive plots revealed two interesting patterns. About half of the participants produced patterns similar to the three exemplary ones in the top row of Figure 4, mostly in a slightly less pronounced form. This pattern is characterized by substantial processing speed benefits in the *COA* = 40 ms and *COA* = 80 ms conditions. Both acceleration of the probe and deceleration of the reference stimulus are present. For the long *COA* = 140 ms condition, the reference rate is increased. As suggested above, this could be a non-specific resource activation coupled with the relative benefit of the reference caused by IOR. However, there is also a strongly increased $v_{cp}^{\mu }$ rate which leads to a broadly shifted psychometric function. The latter hides the disadvantageous effect of the cue at the large COA, which would normally lead to an opposite shift of the psychometric function.

A very different pattern is found in some of the participants (particularly strong in about 20%). Three examples are shown in the bottom row of Figure 4. For these participants, the cue does not exert a beneficial effect on the rates. Most likely, IOR caused by the cue is already present at relatively low COAs. Additionally, no high *v*_*cp*_ rate acts at large COAs to shift the PSS rightwards, as in the pattern described above (Figure 3).

To sum up, as hypothesized, cue–probe categorizations contribute substantially to prior entry. In contrast to the hypothesis, processing rate changes play an important role, too. Another observation is that the cue's encoding rate *v*_*cp*_ also varied between the different COA conditions. Most likely, this is caused by different degrees of interference between target stimuli and cue. Finally, there were substantial individual differences on the subject level. In addition to the main pattern where attention acts beneficially, especially at the medium COAs and suffers from IOR at larger COAs, another pattern was found for a few subjects. For them, the cue always is disadvantageous, lowering the probe rate, and they do not exhibit large rates of cue–probe categorizations that counteract the leftward shift of the psychometric functions.

## Experiment 2

In the previous experiment, the cue led to nonzero estimates of the cue–probe categorization, which contributed to the observed prior entry. In addition, substantial rate changes occurred when the temporal separation of the cue was varied. The second experiment tests whether the rate of cue–probe categorizations is reduced if the spatial distance between cue and probe is increased. That is, it is tested whether the co-locality of cue and probe is crucial for their occasional confusion.

**Figure 6:**
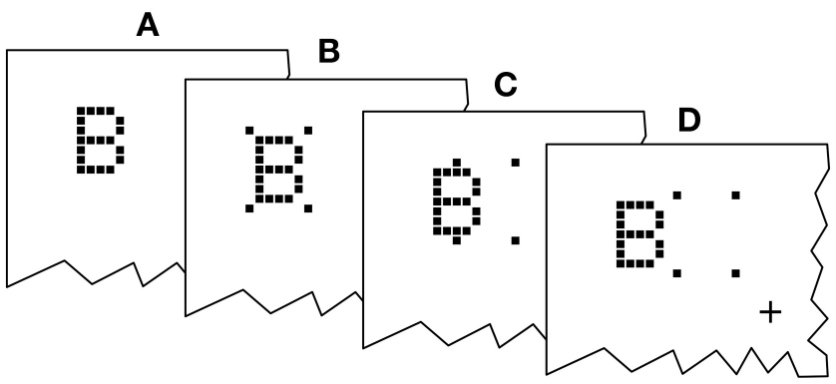
**Illustration of the cue displacement in the second experiment**. **(A)** No-cue condition. **(B)** Cue at the target position. **(C)** Cue shifted halfway (15 px; approx. 0.4°). **(D)** Cue next to the target (60 px; approx. 0.9°). Note that the cue and target are shown here in the same frames for illustration only. In the actual experiment, the cue was always turned off at the moment the target appeared.

For this purpose, the COA was fixed at 80 ms, for which the previous experiment showed cue–probe confusion and a substantial increase of the probe processing rate. The cue was spatially displaced by different offsets (CLD; cue location displacement in pixels) in the experimental conditions. In one cued condition, it was shown at the probe's position, in another condition it was shifted to overlap with the probe, and in a third condition it was displayed completely adjacent to the target (see Figure 6).

### Method

#### Participants

In this experiment, 26 persons participated (17 females, the mean age was *M* = 25.00, *SD* = 9.03). All had normal or corrected-to-normal vision.

#### Stimuli

The stimuli were the same as in Experiment 1.

#### Presentation and response

The general presentation and response procedures were the same as in Experiment 1. The COA was fixed at 80 ms. The cue was displaced by different spatial offsets in the three experimental conditions as shown in Figure 6. A no-cue control condition was included as well. The SOAs and corresponding numbers of repetitions can be found in Table 1 in the “No-cue” and “*COA* = 80 ms” rows. Again, trials from all conditions were presented in an intermixed order.

### Results

The hierarchical Bayesian parameter estimation was similar to Experiment 1.

The model comparison (see Supplementary Material Figure A2B) replicates the pattern from Experiment 1 for the *CLD* = 0 px condition. This is not surprising, because this condition had the exact stimulus presentation sequence as Experiment 1's *COA* = 80 ms condition. In the other conditions, however, the simple TVA-based TOJ model which only allows for attention-modulated changes in probe and reference rates is the best model. This indicates that as the cue is moved away from the target, the cue–probe confusion contribution becomes less important to explain the PSS shift found in psychometric functions.

**Figure 7:**
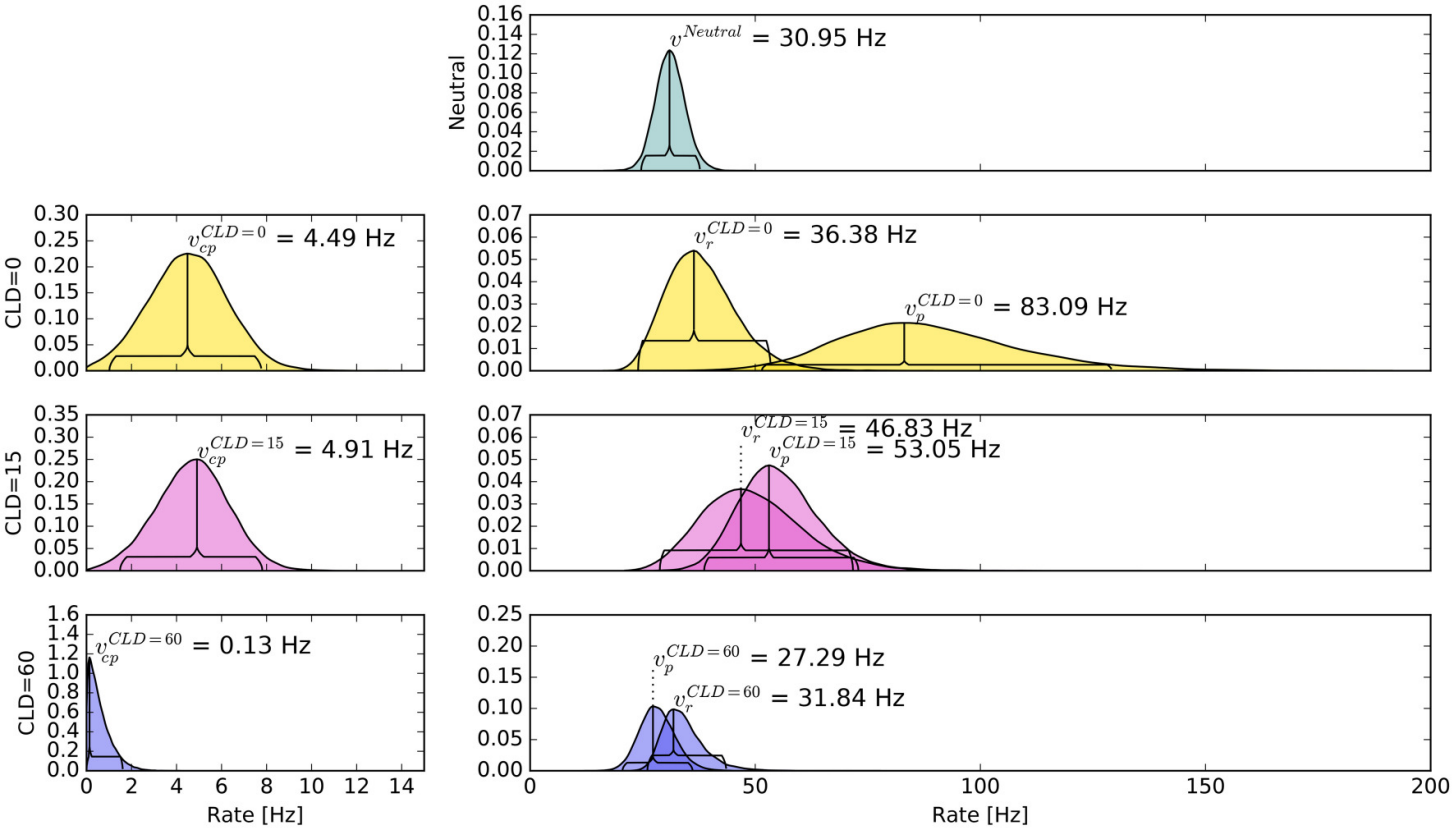
**Bayesian parameter estimates for Experiment 2, visualized in the same way as in Figure 3**. The top row shows the neutral condition, rows two to four show conditions where the cue was displaced according to the CLD (cue location displacement; best viewed in color).

Again group-level posterior density plots were analyzed (leaving out six participants for whom the cue had solely disadvantageous effects; density plots with all subjects are shown in Supplementary Material Figure A4). Figure 7 summarizes the group-level analysis. The processing rate in the neutral condition, *v*^μ^, was estimated at 31 Hz, similar as in Experiment 1. The probe rate $v_{p}^{\mu }=83$ Hz is higher than the reference rate in the *CLD* = 0 px condition. Zero was not within the 95% HDI of the difference [$v_{p}^{\mu }-v_{r}^{\mu }$]; lower boundary at 10 Hz. In the *CLD* = 15 px condition, the probe at 53 Hz was not reliably different from the reference. At *CLD* = 60 px, $v_{p}^{\mu }$ and $v_{r}^{\mu }$ were estimated at 27 and 32 Hz. Both at *CLD* = 15 px and *CLD* = 60 px zero is within the 95% HDI on the rate difference. In short, the processing rate advantage of the probe stimulus decreases with increasing spatial distance of the cue.

The posterior distribution of $v_{cp}^{\mu }$ resembles the corresponding distribution of Experiment 1, *COA* = 80 ms condition. Interestingly, the rate of cue–probe confusions is reduced to virtually zero when the cue is moved so far that there is no more overlap.

**Figure 8:**
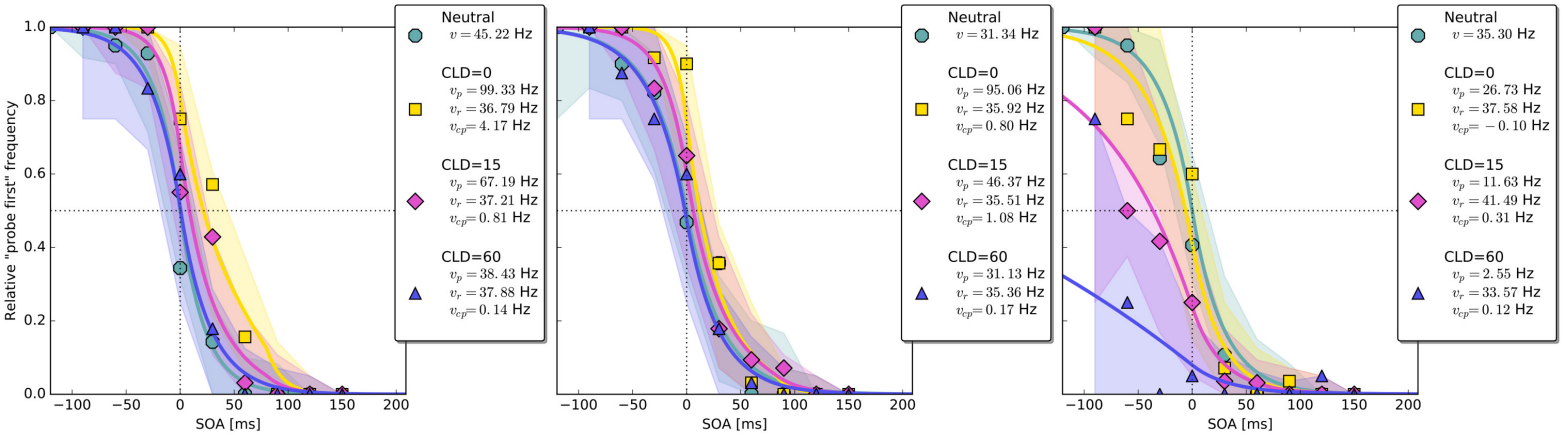
**Exemplary subject-level posterior predictive distributions and raw data plots for the different CLD (cue location displacement) conditions**. Visualization in the same fashion as in Figure 4. The third panel shows an example of the disadvantageous patterns which were excluded in the main group-level analysis (best viewed in color).

The overall pattern can also be seen in the subject-level posterior predictive plots: The cue increases the probe's processing rate most substantially at *CLD* = 0 px. This benefit is reduced at *CLD* = 15 px and almost removed at *CLD* = 60 px (see first two panels in Figures 8). The third panel in Figure 8 shows a pattern similar to those in the lower row of Figure 4, in which the cue is only disadvantageous. The proportion of these two patterns in Experiment 2 is similar to Experiment 1.

### Discussion

As hypothesized, moving the cue spatially away from the target reduced its effect. As in the *COA* = 80 ms conditions in Experiment 1, the cue led to an increase of the probe processing rate. This effect was strongest when the cue was exactly at the probe location. By displacing the cue, the benefit was reduced and virtually removed at *CLD* = 60 px, when the cue was presented at a location next to the target but did not overlap with it. Similarly, the rate of cue–probe confusions was entirely nullified at this CLD.

An interesting difference to the results of Experiment 1 is the size of the cueing effect. The *COA* = 80 ms condition of Experiment 1 and the *CLD* = 0 px condition of Experiment 2 were identical. The cueing effect, however, was substantially larger in the second experiment. Here, the probe rate reaches 83 Hz compared to 69 Hz in Experiment 1. Most likely, cueing is more effective in Experiment 2, because the COA is equal in all cueing conditions, whereas different COAs were used in Experiment 1. The resulting temporal predictability might have strengthened the effect.

In summary, the effective cueing found in the *CLD* = 0 px diminished when the cue was displaced. It did so by effects on the processing speeds of probe and reference and by decreasing the rate of cue–probe categorizations as hypothesized.

## General discussion

In this study, we conducted two cued TOJ experiments and analyzed them with a process-based model. In particular, we tested whether the broad shifts observed for psychometric distributions at large COAs can be caused by sometimes encoding the cue as the probe target. Both experiments showed that such cue–probe categorizations contribute to the shift of psychometric functions. However, they also revealed that substantial changes in the processing rates occur due to the cue. These were advantages at the smaller COAs (40 and 80 ms). At the large COA of 140 ms, IOR occurred, causing a large relative disadvantage for the probe.

These findings are based on a mathematical model of attention in cued TOJs, which was derived from TVA, a general theory of visual attention. In the past 30 years, TVA was successfully applied in many areas (Bundesen et al., 2015; Kyllingsbæk et al., 2015). Furthermore, there is a neural interpretation of the theory which links the cognitive theory to neurophysiology (Bundesen et al., 2005).

The TVA-based TOJ model developed and applied in this article does not require much additional assumptions beyond what follows directly from the basic TVA encoding processes. One example is the notion that the cue also races for being encoded as the probe stimulus. As explained in the Introduction, a cue–probe categorization with a low rate can be expected according to TVA. Due to this firm theoretical basis, we believe that the model captures the essential aspects of cued TOJs. Nevertheless, the following discussion of the results is split into two parts. First, in Section “A Model-based assessment,” those results are discussed whose interpretations necessarily require the described model and its essential correctness. Second, in Section “The coarse patterns in the data,” the results that stand without the model are discussed from an inevitably more general perspective.

### A model-based assessment

#### Effects of the cue processing rates

The cue processing rate *v*_*cp*_ was estimated at relative low values compared to the targets. It was argued above that this rate contributes to the large shifts in psychometric functions. It is interesting to further look into how large this contribution is, because on it hinges the general hypothesis that the cue is sometimes encoded as the probe. How often is sometimes?

The mean rate of cue–probe categorizations, $v_{cp}^{\mu }$, was estimated at 10 Hz for *COA* = 140 ms. With this rate, at negative SOAs the cue would be encoded as the probe in 75% of the trials before being masked by the probe. If one accounts for a commonly sized *t*_0_ of 15 ms, the threshold mentioned in the Introduction, the cue–probe categorization would still happen in 60% of the trials. For participants with widely shifted psychometric functions, *v*_*cp*_ values were around 20 Hz. At such high rates, in 94% (66% when accounting for *t*_0_) of the trials a cue–probe categorization during the COA would succeed.^3^ The strong contribution of probe–cue categorizations to the PSS shift in the *COA* = 140 ms condition can also be seen in Figure 5. Hence, despite their rather low values, the *v*_*cp*_ rates play an important role, especially in accounting for broadly shifted psychometric functions observed in the data of many participants.

### Effects on the target processing rates

According to our hypothesis outlined in the simulation in Figure 1, the COA-modulated effects of peripheral visual cues are explained entirely by the fact that participants sometimes encode the cue as the probe target. As discussed above, these encodings indeed contribute in important ways to explain the data. However, they do not capture the whole story.

In both experiments, we found substantial effects on the processing rates of probe and reference. At *COA* = 80 ms the beneficial effects are most prominent, reflected in the parameter means (see Figure 3, “COA = 80” row). Taking this effective COA and shifting the cue so that only half of it overlaps with the probe reduced the cueing effect on processing rates. Presenting the cue as an entirely separate stimulus next to the probe target (as shown in Figure 6D) virtually removed its effect.

Therefore, in addition to the effects produced when successfully encoding the cue during the COA, its ongoing or incomplete encoding led to the observed influences on the target processing rates by altering the distribution of attention.

In the following, we discuss an explanation based on theoretical assumptions. The main mechanism behind this explanation is the target's ability to seize the low-level resources of the cue if it has only been presented for a short time. If a cue is presented for a longer time, the resources are blocked. To account for all our observations, it is necessary to assume a spatially unspecific alerting effect that makes more resources accessible at longer COAs. All these mechanisms have been discussed in the TVA literature before. The following explanations include the respective references.

**Figure 9:**
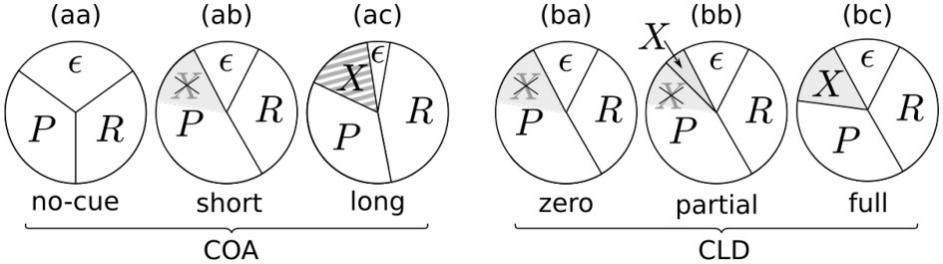
**Potential resource distributions at different COAs (cue onset asynchronies) and CLDs (cue location displacements)**.

The resulting potential resource distributions for varying the COA or CLD are visualized in Figure 9. The pie charts visualize how the resources are divided among the stimuli. Importantly, there is always a partition ϵ of resources which are not distributed to the probe, reference, or cue. The resources ϵ could be involved with encoding extraneous noise (see e.g., Petersen et al., 2010) or be strategically withheld.

Figure 9aa represents a neutral condition. Equal partitions are assigned to the probe (*P*) and reference (*R*). Some amount, as described above, is bound by ϵ. When a cue is shown with a short COA, it has two effects (Figure 9ab): First, the amount of accessible resources is increased, due to a spatially non-specific alerting effect of the cue. This can be seen in the reduction of the ϵ partition, which increases the effectively available resources *C*, the sum of all concurrent stimulus rates. Increases in TVA parameter *C* caused by alerting signals have been reported before (see Matthias et al., 2010).

The second mechanism to explain effects at short COAs is the idea that the probe can seize the low-level resources already activated by the cue. Therefore, it has an attentional benefit. This reuse of cue resources by a target was already hypothesized in Tünnermann et al. (2015) and described with TTVA mechanisms (temporal theory of visual attention; Petersen et al., 2012, 2013). In effect, the probe can be processed at a higher rate than the reference, as we found most prominently in the *COA* = 80 ms condition (see Figure 3), but also in the *COA* = 40 ms condition for several individuals in Experiment 1 (see Figure 4).

For larger COAs, as shown in Figure 9ac, ϵ is further reduced. That is, even more resources are available to encode the cue and targets. However, due to the long COA, encoding the cue has progressed so far that high-level neurons which represent the cue have already been tied via feedback loops to the low-level resources. These lock the resources, as suggested by Petersen et al. (2012) under similar conditions for the attentional dwell-time paradigm. In the dwell-time paradigm, the locking effect is strongest at intervals of 100–200 ms. In our setting, *R* now is a greater partition than *P*, and the reference is encoded at a higher rate, as we found in the *COA* = 140 ms condition of Experiment 1. This is well in agreement with the timing in the experiments by Petersen et al. (2012).

The chart in Figure 9ba corresponds to the *CLD* = 0 px condition of Experiment 2. Because this condition matches the *COA* = 80 ms condition of Experiment 1, it has the same resource partitioning as seen in Figure 9ab.

Because the COA was not varied in Experiment 2, the ϵ partition is equal in all cases (Figures 9ba–bc). What varies is the portion of preactivated resources which the probe can seize from the cue. If the cue and target positions overlap entirely, the probe inherits all resources preactivated by the cue (Figure 9ba). If it overlaps only partially, the probe can capture only some of the cue's resources (Figure 9bb). If it is presented at a completely separate position (Figure 9bc), the probe cannot seize any resources preactivated by the cue and is therefore processed with the same resources as in the neutral condition. Figures 9bb,bc includes an additional partition, *X*, which represents the resources activated by the cue that cannot be captured by the probe. Most likely, these would be involved with encoding the cue as a separate stimulus, because it becomes easier to recognize with growing CLD. The rate *v*_*x*_ is unobservable in the present cued TOJ paradigm because encoding the cue at a separate position is assumed to not influence the TOJ. Because the VSTM capacity is usually three to four items, it could be encoded without interfering with the probe and reference targets.

To summarize, in our TVA-based TOJ model, the large shifts of psychometric distributions induced by a cue originate from two mechanisms. There are contributions from occasionally encoding the cue as probe. In addition, the processing rates of probe and reference targets are changed by the cue. At small COAs, these effects benefit the probe. At the large 140 ms COA, an inhibitory mechanism leads to the reference stimulus being processed at a higher rate than the probe stimulus. This would shift the psychometric distributions leftwards. However, it is compensated—even overcompensated—by the aforementioned categorizations of the cue. Ultimately, this interplay produces the broadly rightward shifted distributions which before could only be modeled by introducing a large artificial delay parameter that lacks theoretical justification (see Tünnermann et al., in press).

A qualitative posterior predictive assessment (see Kruschke, 2013) on the subject level revealed that the model fits the data well. The only discrepancy that may be systematic can be seen in the second and third plot in the upper row of Figure 4. The course of the curves appears to miss some of the high data points at SOAs 30 and 60 ms. Possibly, the fit could be improved in this regard by allowing for a small additional delay that would shift the distribution rightwards. Importantly, “small” refers to a magnitude that is theoretically plausible for a potential *t*_0_ difference (Tünnermann et al., in press) of 20–30 ms. As a consequence, the slope of the distribution at the large COA would get steeper, which would be reflected in a higher probe rate. Hence, most likely, the current model slightly overestimates the shift of resources in favor of the reference stimulus at the 140 ms COA. This will be further investigated in future work.

Future models should also aim at including the subject-level patterns that show a purely disadvantageous cueing effect. In preliminary tests, including a rate associated with the cue that leads to “reference first” judgments (as opposed to “probe first” resulting from *v*_*cp*_) did improve the fit of the hierarchical model. Such a parameter could be understood as a rate with which the cue is recognized and its location ignored, leading to a judgment in favor of the reference stimulus at the other location. The parameter indeed led to improved fits. However, stronger assumptions about all parameters (via more informative priors) had to be made. Furthermore, such a parameter lacks a theoretical justification that goes beyond the *ad-hoc* mechanism described above and should be compared to similar alternatives, as for example probabilities for occasional lapses.

### The coarse patterns in the data

Independently of the model-dependent findings described above, certain patterns in the data are of a rather general nature. This means that certain conclusions can be drawn without assuming the formal model.

In Experiment 1, several participants showed large cue-induced rightwards shifts of psychometric distributions (see Figure 4). Some participants, however, produced a pattern in which increasing COAs gradually shift the psychometric distributions leftwards and weaken their slopes. In the usual psychophysical analysis, this leftward shift represents a relative advantage of the uncued stimulus over the cued one. The weaker slope corresponds to a decline in discrimination performance. Taken together, these effects speak for a disadvantageous effect of the cue on the probe stimulus, just as in our model-based assessment.

Intriguingly, the same disadvantageous pattern is found in the subject-level plots of some participants in Experiment 2. One example is shown in Figure 8 (right panel). Therefore, it stands to reason that the cue becoming a clearly distinct visual event, either because of temporal separation (Experiment 1) or by spatial separation (Experiment 2), is the underlying cause of this effect. Both temporal and spatial distances reduce the masking of the cue by the probe, increasing its visibility. Possibly, the emergence of a separate stimulus interferes by constituting additional load for the visual system. Also, eye movements could be discussed as the origin of the disadvantageous rate pattern because they were not controlled for. Usually, such control is unnecessary because programming and executing saccades takes too long to be a useful strategy in TOJs and therefore it can be assumed that participants refrain from performing any gaze strategies (see Tünnermann et al., 2015). This argument, however, is hinged on the observation that participants are successful at TOJs or possibly biased in the usual direction. This is not the case for the participants who show the disadvantageous pattern in the current experiments. Their low count of “probe first” judgments, even at the largest negative SOAs, might be the consequence of disadvantageous gaze strategies. In this view, the question then boils down to why they occur in these experiments so frequently compared to earlier studies. The relatively large temporal and spatial distances between probe and cue, together with the enhanced visibility, might motivate or involuntarily trigger participants to perform eye movements.

Furthermore, independent of any model, the data patterns of different participants vary substantially. They seem to depend on how the presentation parameters interact with the timing and resources of the individuals. The emergence of disadvantageous and advantageous cuing effects in different subjects is a striking example. Therefore, assessing the results of only a very few participants, as sometimes done in traditional psychometric research, may make it difficult to capture the differential impact of attentional factors. Similarly, averaging the raw judgment counts over participants and fitting the summarized data would average out shape alterations of the psychometric distributions that carry important information. In the present study, we used a hierarchical Bayesian approach to avoid such problems. Parameters estimates are available on the subject-level (with outliers subdued by the desirable effect of shrinkage, see, e.g., Kruschke and Vanpaemel, 2015). Also, group-level estimates provide an overview of the probabilities to find certain values in the population.

## Conclusion

In the present study, we conducted two experiments to test whether occasionally encoding the cue as the probe target in TOJs drives the commonly observed attention effect. In typical psychometric approaches (e.g., Shore et al., 2001), the attention effect is quantified as a shift of the PSS. In previous TVA-based analyses, these broad shifts were captured with an additional delay parameter which has no theoretical justification (Tünnermann et al., in press).

The present results indicate that these patterns in psychometric distributions can be accounted for by an interplay of different mechanisms which are associated with encoding the presented stimuli. Important contributions come from occasionally encoding the cue as the probe target. This is estimated to happen in more than 90% of the *COA* = 140 ms trials for participants who show particularly broad shifts in the PSS.

In addition to influences of cue–probe confusions on the judgments, we observed influences on the processing speed of probe and reference targets exerted by the cue. These can be regarded as “genuine attention effects,” as alterations in the resource distribution induced by the cue speeding up or slowing down subsequent processing.

The interplay of these two types of influences on temporal-order judgments leads to the observed psychometric distributions. According to our model, the genuine attention effect first rises, peaking probably somewhere around the 80 ms COA, and then decays. At 140 ms, it is even strongly overruled by inhibitory effects cased by the cue.

These genuine attention effects agree better with results from reaction time and accuracy experiments with location cueing than earlier cued TOJ studies that quantiefied attention effects via how far the PSS is shifted. In the location cueing experiments, the beneficial effects peak rather early (around 100 ms or earlier, for a summary see Wright and Ward, 2008, Chapter 2) and decline afterward. Eventually IOR is evoked (Posner and Cohen, 1984; Klein, 2000). Earlier TOJ studies described effects that peak later. These experiments also show a decline of the effect after peaking; however, it never inverses, as would be expected due to IOR. Scharlau et al. (2006) suggested several reasons why they “observed facilitation at the primed location even for the longest priming SOA, instead of inhibition of return (IOR; Klein, 2000),” including that IOR may be weakly present and overruled by facilitation or absent in presentations containing sequential stimuli at multiple locations. An alternative explanation offered by the current study is that the shift of psychometric distributions is not a direct measure of genuine attention effects. As suggested above, IOR may be at work biasing the resource distribution in favor of the reference stimulus as visualized in Figure 9ac. The absence of IOR footprints when measuring the shift of psychometric functions is then due to cue–probe categorizations, which conceal and overcompensate the inhibitory rate effects.

The results of the present study imply interactions between genuine attention effects, cue–target confusions on a perceptual level, and IOR, which conjointly lead to prior entry. What is the evidence for such interactions outside the realms of prior entry? As already mentioned, cueing studies typically find time courses that match those implied by the genuine target processing rate effects we find. The possibility that such effects combine with IOR was discussed, for example, by Klein (2000), see Box 1 in his article. Substantiating this, there exists evidence that IOR is due to a concurrent non-attentional process, as demonstrated by Zhao and Heinke (2014). However, there is no evidence for something analog to cue–target confusions outside the TOJ domain. In cued TOJs, the cue's onset is an important target feature, due to the temporal judgments which are required. In other paradigms, this may be less so, for example, if reaction time or target discrimination is used to measure attention. Furthermore, in conventional cueing experiments with reaction time measurement, cue–probe confusions will either lead to errors or prolonged responses and are thereby taken into account. By contrast, in TOJs, they exaggerate the cueing effect by largely shifting the PSS, which in earlier studies was confused with genuine attention effects that alter target processing speed.

What do the results of the present study imply for attention research over and above prior-entry studies in TOJs? In the field of selective visual attention there is some habit of dichotomizing potential processes which are assumed to produce prominent attention effects. Examples are distinctions such as exogenous vs. endogenous attention, early vs. late selection, or peripheral vs. central cues (e.g., see Carrasco, 2011). Such dichotomies may indeed be helpful to describe what mainly drives certain effects or what solely drives them in very simple conditions. However, as noted for example by Ansorge and Heumann (2003) in the context of peripheral vs. central cueing, they may not be sufficient to describe the variable spectrum of observations and thus need some revision. In this line of thinking, Bundesen (1990) has dissolved the early vs. late selection dichotomy and provides an approach that captures essential aspects of both. Similarly, the top-down vs. bottom-up dichotomy was declared inadequate (e.g., Awh et al., 2012). The present study provides implications in a similar direction. To uncover conspicuities such as the time course of PSS shifts in TOJs, the distinction between cues and targets, which is present in many other paradigms as well, needs to be countermanded in its strict form. The cue must be considered a full stimulus in its own right. It may influence the result not only by directing attention toward its location, but also by its encoding. In the present study, the cue's influence was exerted entirely via cue–target confusions, which are likely in some tasks. A similar route of influence is provided by the resources required to process the cue. In the TVA framework, its share of the processing resources and the resources it occupies in VSTM can and should be modeled, too, in future work. In summary, peripheral cues should be demoted from their special status as explicit attention-guiding entities. They should rather be modeled as ordinary stimuli, which can direct attention and affect the response due to their processing.

To conclude, the proposed mechanisms explain the influence of peripheral cues on a fine-grained process-based level. In future work, the influence on the resource distribution should be further tested. It is plausible because it was derived from TVA including the recent TTVA extensions of the theory into the temporal domain. A weakness of the current explanation is, however, that it is based on the assumption of changes in quantities that are unobservable in the present paradigm. Such “dark rates”—to borrow an image from cosmology—*v*_ϵ_ and *v*_*x*_ are involved with processing that does not directly interact with TOJs (cf. Figures 9ab–bc). In future experiments, however, they could be controlled or even measured. For instance, if the box cue is replaced by a postmasked letter, the rate *v*_*x*_ could be estimated as in typical TVA-based letter report experiments. Then it could be tested if the model prediction is true that “*v*_*p*_ + *v*_*r*_ at zero CLD” equals “*v*_*p*_ + *v*_*r*_ + *v*_*x*_ at full CLD,” which follows from Figures 9ba–bc.

## Author contributions

Conceived the experiments: JT, IS. Implemented and performed the experiments: JT. Simulated, modeled, and analyzed the data: JT. Wrote the initial manuscript: JT. Supervised the generation of the manuscript: IS. Revised the paper: JT, IS.

### Conflict of interest statement

The authors declare that the research was conducted in the absence of any commercial or financial relationships that could be construed as a potential conflict of interest.

## Footnotes

^1^Note that a subset of the participants showed a disadvantageous effect caused by the cue which is reflected by a negative PSS. This interesting pattern is discussed later. This study set out to explain the strong positive PSS shifts typically observed (and observed in the majority of our participants). Therefore, ten participants with disadvantageous effects were excluded from the group-level analysis here. Group-level posterior densities with all participants can be found in Supplementary Material “Group-level estimates including all participants.”

^2^Rate differences across conditions should be considered only with reservation. All conditions were estimated in independent samplings, and the differences and their certainties across conditions cannot be estimated. Small apparent differences in some conditions may not be statistically significant.

^3^For positive SOAs, the percentages would be slightly less than the calculated values, because the reference stimulus could occasionally be encoded between cue and probe, terminating processing.

## Supplementary material

The Supplementary Material contains an appendix to the article covering the technical details of the model and further analyses for this article. It can be found online at: http://journal.frontiersin.org/article/10.3389/fpsyg.2016.01442
