# Supplementary material for: Peripheral Visual Cues: Their Fate in Processing and Effects on Attention and Temporal-Order Perception
Source: Front Psychol. 2016 Oct 6;7:1442. doi: 10.3389/fpsyg.2016.01442 (PMC5052275; doi:10.3389/fpsyg.2016.01442)
Supplement: Supplementary file 1 [file Appendix.pdf]

APPENDIX

Appendix to: Tünnermann J and Scharlau I (2016). Peripheral Visual Cues: Their Fate in Processing and Effects on Attention and Temporal-order Perception. Front. Psychol. 7:1442. doi:10.3389/fpsyg.2016.01442

Deriving Probe First Probabilities for Cued TOJs from TVA

According to TVA, the probability of encoding a single stimulus x until a point in time t is given as $1 - exp((t - t_0) \cdot v_x)$, when $t > t_0$. Otherwise, it is 0. Parameter t_0 is a delay before the race for VSTM encoding starts (see Bundesen, 1990, for details) and is omitted here.[4] Parameter v_x is the rate at which stimulus x is processed. According to our hypothesis, encoding probe (rate v_p) or cue (as the probe, rate v_{cp}) before the reference stimulus (rate v_r) leads to a "probe first" response.

In the following, the probability for reporting "probe first" is derived from these encoding events. If the SOA is negative or zero, this probability is given as

$$\begin{aligned} P(p^{1st}|v_p, v_r, v_{cp}, SOA, COA) = \\ & P(T_{cp} < COA) + \\ & (1 - P(T_{cp} < COA)) \cdot (\\ & \quad P(T_p < |SOA|) + \\ & \quad\quad (1 - P(T_p < |SOA|)) \cdot P(T_p < T_r) \\ &), \qquad \text{if } SOA \leq 0, \end{aligned} \tag{A1}$$

where the probability that the cue is encoded as probe before any other stimulus appears, $P(T_{cp} < COA)$, is $1 - exp(-vc \cdot COA)$. If it was not encoded during this time, with probability $(1 - P(T_{cp} < COA)) = exp(-v_{cp} \cdot COA)$, the probe is encoded during the SOA with probability $P(T_p < SOA) = 1 - exp(-v_p \cdot |SOA|)$. If this is not the case, with probability $exp(-v_p \cdot |SOA|)$, probe and reference race together. Then, the probe finishes first with $P(T_p < T_r) = v_p/(v_p + v_r)$, according to Luce's choice axiom (see e.g., Luce, 1977).

If the SOA is positive but smaller than the COA, the probability of judging probe first can be calculated as

$$\begin{aligned} P(p^{1st}|v_p, v_r, v_{cp}, SOA, COA) = \\ & P(T_{cp} < (COA - SOA)) + \\ & (1 - P(T_{cp} < (COA - SOA))) \cdot (\\ & \quad P(T_{cp} < T_r \text{ during } SOA) + \\ & \quad (1 - P(T_{cp} < SOA)) \cdot (1 - P(T_r < SOA)) \cdot \\ & \quad\quad P(T_p < T_r) \\ &), \qquad \text{if } COA \geq SOA > 0, \end{aligned} \tag{A2}$$

where $P(T_{cp} < COA - SOA)$ is the probability that the cue is encoded as the probe before any other stimuli are shown, calculated as $1 - exp(-v_{cp} \cdot (COA - SOA))$. If no encoding event happened, with probability $(1 - P(T_{cp} < (COA - SOA))) = exp(v_{cp} \cdot (COA - SOA))$, encoding the cue as probe during the SOA and before the reference is encoded, contributes with $P(T_{cp} < T_r \text{ during } SOA) = v_{cp} \cdot (1/(v_{cp} + v_r) - exp(-v_{cp} \cdot SOA - v_r \cdot SOA)/(v_{cp} + v_r))$. If no encoding events occurred during the SOA, with probability $(1 - P(T_{cp} < SOA)) \cdot (1 - P(T_r < SOA)) = (exp(-v_{cp} \cdot SOA) \cdot exp(-v_r \cdot SOA))$, the probe wins racing the reference with $P(T_p < T_r) = v_p/(v_p + v_r)$ (see previous paragraph).

Finally, if the SOA is positive but larger than the COA, the calculation is as follows:

$$\begin{aligned} P(p^{1st}|v_p, v_r, v_{cp}, SOA, COA) = \\ & (1 - P(T_r < (SOA - COA))) \cdot (\\ & P(T_{cp} < T_r, \text{ during } COA) + \\ & \quad (1 - P(T_{cp} < COA)) \cdot (1 - P(T_r < COA)) \cdot \\ & \quad\quad P(T_p < T_r) \\ &), \qquad \text{if } SOA > COA > 0, \end{aligned} \tag{A3}$$

where $(1 - P(T_r < (SOA - COA)))$ is the probability that the reference is not encoded before any other stimuli are shown. Because no cue was shown yet, a rate amounting to half of all available resources is considered in this probability, $exp(-((v_r + v_p + v_{cp})/2) \cdot (SOA - COA))$. During the COA, the cue is encoded as probe before the reference is encoded with probability $P(T_{cp} < T_r, \text{during } COA) = v_{cp} \cdot (1/(v_{cp} + v_r) - exp(-v_{cp} \cdot COA - v_r \cdot COA)/(v_{cp} + v_r))$. If no encoding events occurred during the COA, with probability $((1 - P(T_{cp} < COA)) \cdot (1 - P(T_r < COA))) = (exp(-v_{cp} \cdot COA) \cdot exp(-v_r \cdot COA))$, the probe wins racing the reference with $P(T_p < T_r) = v_p/(v_p + v_r)$, as explained above.

A Hierarchical Bayesian Model of Cued TOJs

For every participant, the distribution of "probe first" judgments over the SOAs was determined for each condition. These counts, SOAs, and their repetitions, of all participants were submitted to a Bayesian hierarchical parameter estimation (Kruschke & Vanpaemel, 2015) and a model comparison (see following section). We used a Hastings-Metropolis sampler implemented in PyMC3 (Salvatier, Wiecki, & Fonnesbeck, 2016). Sampling start points were selected using PyMC3's find_MAP function with a variant of Powell's (1964) optimization method. For most conditions, 200000 samples were drawn. In some conditions, especially within the model comparison reported in the next section, convergence was slower and more samples had to be drawn (4×10^6 samples in the $COA = 140$ ms condition for the full model, and in the $COA = 80$ and $COA = 140$ conditions of the (v, v_{cp}) model, and for the $COA = 80$ ms condition for the (v_p, v_r) model; 5.6×10^6 samples in the $COA = 140$ ms condition for the (v_p, v_r) model).

Such a hierarchical model estimation has the advantage that both group-level and subject-level parameter estimates can be obtained. We described this approach for TVA-based TOJ models in Tünnermann et al. (in press) and provide a brief outline of the sampling model here.

The Bayesian estimation procedure requires a generative model. Random variables are sampled from appropriate distributions. The

[4] In our earlier simple TVA-based TOJ models (Tünnermann et al., in press), we omitted t_0 because it is theoretically equal for probe and reference, and does not alter their VSTM arrival. Here, in the more complex model of cued TOJs we omit it again, as the argument above holds for most parts of the model. Note, however, that omitting t_0 indeed could be a simplification with respect to the processing of the cue and the time of resource allocation.

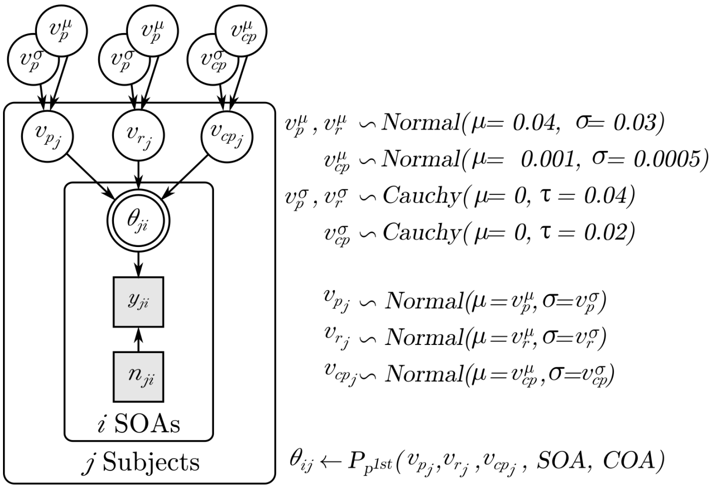

FIGURE A1 | Graphical model describing how the "probe first" count y_{ji} and repetitions n_{ji} relate to the parameters. The "~" refers to the sampling of random variables, the "←" represents the deterministic evaluation of the order judgment function. See Lee and Wagenmakers (2014) for further details on the notation. Note that the subject-level rates ($v_{p\ j}$, $v_{r\ j}$, $v_{cp\ j}$) were obtained using corresponding non-central parametrizations for more effective sampling (see Papaspiliopoulos, Roberts, & Sköld, 2003). The unit of SOAs (stimulus onset asynchronies) is milliseconds, therefore the unit of the rate parameters and their priors is elements per millisecond. Results reported in the article have been multiplied with 1000 and are therefore in Hz.

relations between them is shown in **Figure A1**. For our TOJs, at each SOA i, the responses originate from a binomial distribution with a success rate θ_i and the repetitions of SOA i. The success rate θ_i is calculated based on Equations A1–A2 described in the previous section. That is, θ_i depends on the "probe first" c ounts, the SOA, and the COA, which are input data and the rate parameters which are estimated. In addition to the probe and reference rates, v_p and v_r (as for example used in Krüger, Tünnermann, & Scharlau, 2016), v_{cp}, the rate of cue–probe categorizations are included as well.[5]

For every participant, the subject-level rate parameters are drawn from group-level normal distributions with means v^μ and standard deviation v^σ, for $v = \left[v_p, v_r, \text{and } v_{cp}\right]$. The prior distributions are shown in **Figure A1**. These priors were weakly informed by dif-ferent sources. They were chosen taking into account the rough range in which TVA parameters vary in general, what we observed in earlier analyses, and theoretical considerations. In particular, the tighter prior on v_{cp} group parameter is motivated by the fact that the participants were not asked to report the corresponding stimu-lus. According to TVA, the pertinence values should then be much lower than that for targets. Furthermore, the simulations showed that v_{cp} rates in the range of target rates lead to implausible shifts in the PSS beyond the COA.

These priors keep the informativeness weak enough to let the data determine the result. Most importantly, the same priors were used for all conditions. Therefore, any differences between the experimental conditions originate from the data.

Model Comparison

The full model which was used to evaluate the experiments of this study was compared to two simpler alternatives. The full model (v_p, v_r, v_{cp}) contains parameters for attention-modulated rate effects and the cue–probe confusion rate. One simpler version (v_p, v_r) does not include the cue–probe confusion rate and corresponds to the simple TVA-based TOJ model used in earlier studies (Krüger et al., 2016; Tünnermann et al., in press). The other simpler alternative (v, v_{cp}) has only one main processing rate for both the probe and reference stimulus and the cue–probe confusion rate. Hence, in this version, any shift of the PSS is caused by cue–probe confusions. To compare the models, the WAIC (Watanabe-Akaike information criterion, see, e.g. Gelman, Hwang, & Vehtari, 2014) was computed for the different attention conditions using the PyMC3 library (Salvatier et al., 2016).

The results of the comparison are shown in **Figure A2**. For Experiment 1 (**Figure A2A**), the full model is the best in all conditions (lowest WAIC score). It is followed by the simple model (v_p, v_r) in the conditions with COAs of 40 and 80 ms. In the 140 ms COA con-dition, where the largest PSS shifts are observed, the simple model (v_p, v_r) shows the worst performance.

For Experiment 2 (**Figure A2B**), the pattern is replicated in the $CLD = 0$ px condition, which had the same stimulus presentation as the $COA = 80$ ms condition from Experiment 1. For the other conditions, the simple model (v_p, v_r) shows the best performance. As the cue is moved away from the target with increasing CLD, the additional cue–probe confusion rate apparently become less important, leaving the simple model a sufficient description of the data.

[5]Equation 1 with parameters v_p, v_r, and v_{cp} is only used for the attention conditions. For the neutral conditions, a simpler form with only one rate $v = v_p = v_r$ is used, as in Tünnermann et al. (in press).

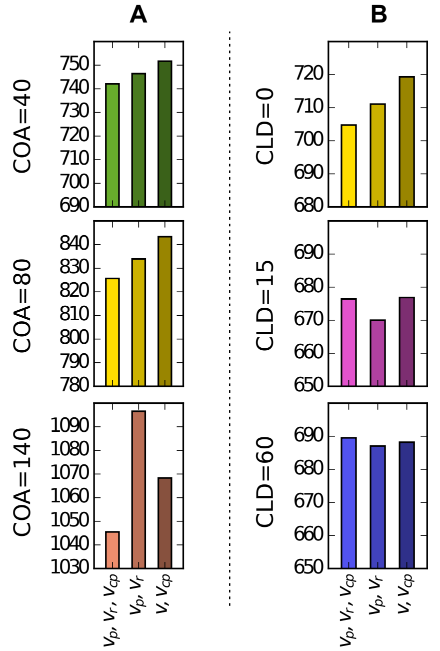

FIGURE A2 | WAIC (Watanabe-Akaike information criterion). Scores for the different conditions from **A** Experiment 1 and **B** Experiment 2. Lower scores reflect higher predictive accuracy.

Group-level Estimates Including All Participants

In the discussion of the experimental results, group-level estimates were shown which had been calculated excluding participants which showed disadvantageous cueing effects. The following figures show the group-level estimates including all participants. Importantly, these distribution must account for both the beneficial effect of cueing found in most participants and the disadvantageous effect found in some. Therefore, the patterns are less clear but in principle the same as the ones discussed in the main part of the article.

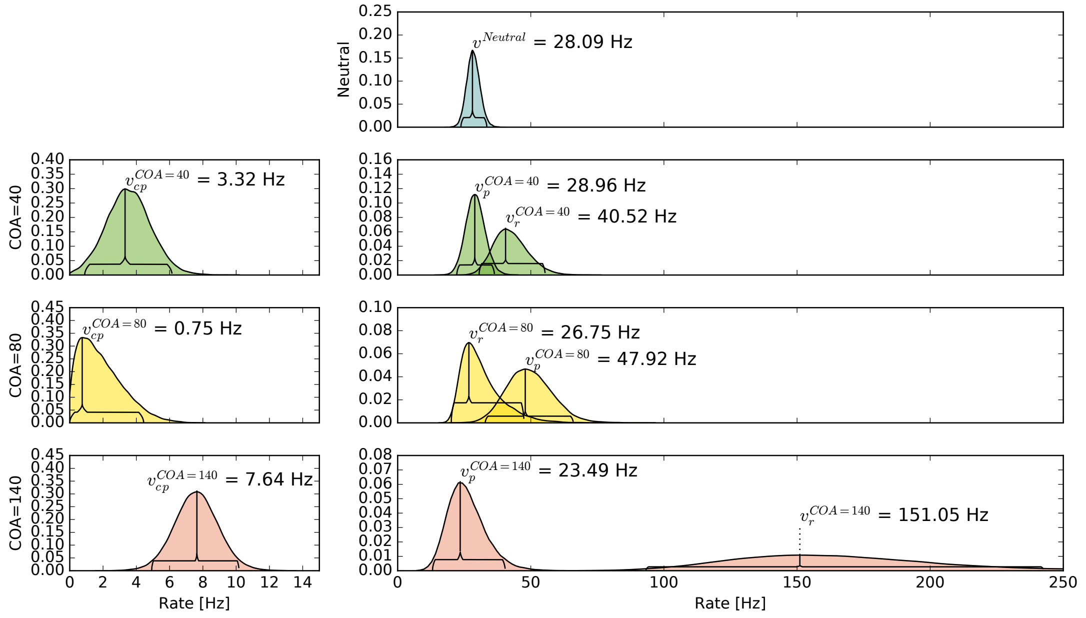

FIGURE A3 | Bayesian parameter estimates including all participants of Experiment 1. Each row contains group-level posterior density plots of the rate parameters *v* from the different COA (cue onset asynchrony) conditions. Horizontal curly brackets mark the ranges of the 95 % highest density intervals (best viewed in color).

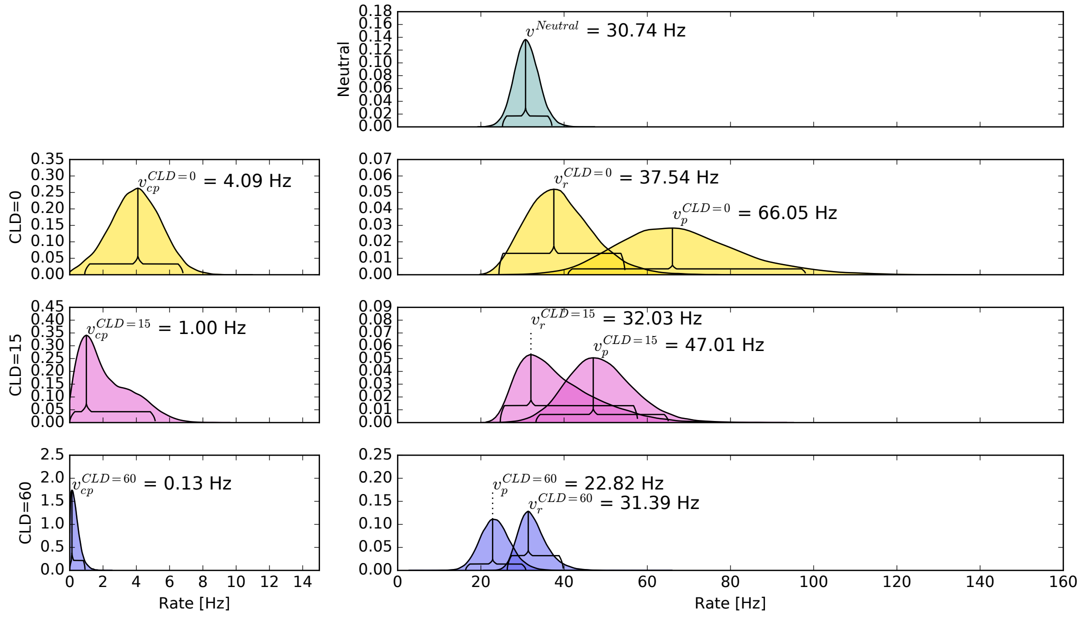

FIGURE A4 | Bayesian parameter estimates including all participants of Experiment 2. Each row contains posterior density plots of the rate parameters *v* from the different COA (cue onset asynchrony) conditions. Horizontal curly brackets mark the ranges of the 95 % highest density intervals (best viewed in color).
